# The Hypothesis of Connecting Two Spinal Cords as a Way of Sharing Information between Two Brains and Nervous Systems

**DOI:** 10.3389/fpsyg.2017.00105

**Published:** 2017-01-31

**Authors:** Amílcar Silva-dos-Santos

**Affiliations:** ^1^Department of Psychiatry, Hospital Vila Franca de XiraVila Franca de Xira, Portugal; ^2^Institute of Pharmacology and Neurosciences, Faculty of Medicine, University of LisbonLisbon, Portugal; ^3^Unit of Neurosciences, Institute of Molecular Medicine, University of LisbonLisbon, Portugal

**Keywords:** spinal cord stimulation, brain-to-brain interface, spinal cord – spinal cord connection, spinal cord – spinal cord interface

## Abstract

Direct communication between different nervous systems has been recently reported through Brain-to-Brain-Interfaces and brainet. Closed loops systems between the brain and the spinal cord from the same individual have also been demonstrated. However, the connection between different nervous systems through the spinal cord has not yet been considered. This paper raises the hypothesis that connecting two spinal cords (spinal cord – spinal cord connection) is an indirect mean for communication of two brains and a direct way of communication between two nervous systems. A concept of electrical fingerprint of a drug is introduced. The notion of connection between two parts of the same spinal cord to treat a paraplegic patient is also introduced. Possible applications of this technique are discussed in the context of psychology, psychiatry and mental health. Also, it is discussed that external information injected to a spinal cord as well as spinal cord – spinal cord connection can become new tools to (1) study the physiology of the nervous system, (2) model specific behaviors, (3) study and model disease traits (4) treat neuropsychiatric disorders and (5) share information between two nervous systems.

## Introduction

In February 2013, the notion of Brain-to-Brain-Interface was introduced in a pioneering work reporting that a brain-machine interface can be applied to share real-time sensorimotor information between the brains of two rodents performing a cognitive task (Pais-Vieira et al., 2013). Soon after, other works reported similar findings such as the sharing of hippocampus representations between two rodents (Deadwyler et al., 2013), the transmission of visual information between a human and a rodent (Yoo et al., 2013) and the transmission of motor information between two humans (Grau et al., 2014; Rao et al., 2014; Stocco et al., 2015).

In 2015, it was demonstrated that it is possible to connect the brain of more than two animals creating what was coined brainet (Pais-Vieira et al., 2015; Ramakrishnan et al., 2015). It has been demonstrated that it is feasible to form a closed loop for artificial communication between a brain of a primate and its spinal cord (Zimmermann and Jackson, 2014; Capogrosso et al., 2016). However, the communication between the nervous system of two different animals through a connection of the spinal cords has not been considered. The hypothesis presented in this paper was inspired, in part, by the published work and protocol regarding spinal cord stimulation to treat epilepsy induced with pentylenetetrazol (PTZ; Pais-Vieira et al., 2016), the papers and protocols of brain-to-brain interface (Pais-Vieira et al., 2013), brainet (Pais-Vieira et al., 2015; Ramakrishnan et al., 2015) and also by the application of spinal cord stimulation to treat rodents models of Parkinson’s Disease (Fuentes et al., 2009).

## The Hypothesis

This paper proposes the hypothesis that it might be possible to share information between the nervous system of two rodents through the connection of the spinal cords.

## The Evaluation of the Hypothesis

**FIGURE 1:**
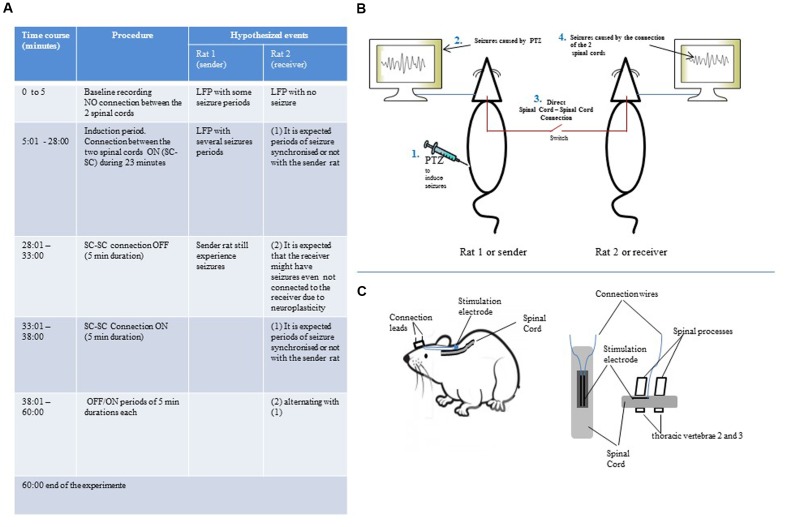
**(A)** Experiment protocol for spinal cord – spinal cord connection showing periods of baseline recording, induction as well as periods of 5 min each of connection ON/OFF. **(B)** Illustration of the setup for LFP recording. **(C)** Illustration of the configuration of the spinal cord electrode.

To test the hypothesis, adults Long Evan rats weighing between 250–350 g will be used. Each rat will be implanted with both brain electrodes and spinal cord electrodes. The brain electrodes will be implanted in the right M1 and left S1 areas according to the procedure described in previous works (Pais-Vieira et al., 2016). These electrode aims to record the Local Field Potential (LFP; Lin and Gervasoni, 2011) as well as single unit activity (Wiest et al., 2008). The spinal cord electrode will be implanted into the epidural space of the dorsal column of thoracic vertebrae 2 (T2) as described by Yadav et al. (2014). As each spinal cord electrode has two leads, the connection cable will consist of two wires that will be plugged to each lead. This cable will have a switch to establish or interrupt the connection between the two spinal cords. The signal to be transmitted or shared between the two rats will be seizure activity provoked by the injection of Pentylenetetrazole. The procedure to induces seizure with PTZ is described elsewhere (Pais-Vieira et al., 2016). Besides the electrical recording of LFP and single units, the seizure activity and the behavior of the animals will also be monitored using a video recording system. The protocol for the connection of the two spinal cords is summarized in **Figures 1A–C**. After injecting the sender rat with intraperitoneal PTZ, the simultaneous LFP and video recording session of the two rats will begin. However, during the first 5 min, no connection between the two spinal cords will be made to allow a baseline recording where the sender rat will have seizure episodes and the receiver rat will not experience seizures. Following this period, the switch of the connection cable will be turned on to establish the connection between the two spinal cords. It will be created an induction period of about 23 min to allow a free communication between the two nervous system. After this time, the connection will be switched off for 5 min. After that, it will follow an ON/OFF periods of 5 min each meaning that the connection will be established during 5 min and then interrupted during 5 min. The total duration of this experiment will be 60 min counting from the beginning of the baseline recording. During the whole experiment, the seizure activity will be recorded in video, single units and LFP.

## Discussion

### The Receiver Rat Will Have Seizures after the Connection of the Two Spinal Cords

After the first connection of the two spinal cords, it is expected that the receiver rat will develop seizures after few seconds/minutes. This seizure would be caused by the electrical information that codifies the seizures induced by the chemical substance PTZ. This can show that the spinal cord can be a way to send codified electrical information from an external source to the cortical region of a receiver. It may also indicate that the artificial bridge between two spinal cord will be possible even with a direct connection without amplifiers.

### Once Induced, the Receiver Rat Will Experience Seizures Episodes even during the Disconnected Periods

One can speculate that this would be possible due to the property that neuronal network has to memorize and reproduces repeated electrical activity and also due to neuroplasticity. The fact that the information is coming from an external source would not be an exception to this neurophysiological phenomenon (the source of neuronal code can be of any kind, either from another animal – such as in this case – or from a computer for example). This can be a mechanism with physiological applications such as (1) modeling and studying of stimulus codifying neurophysiological mechanisms or behavior that can be injected into the nervous system through the spinal cord; (2) modeling of disorders: an information that codifies a particular trait of disease can be injected into the nervous system via the spinal cord.

### The Flow of Neural Information in One Direction from a Nervous System to Another or Sharing of Information between Two Nervous Systems?

This paper presents the notion that the receiver rat will experience seizures coming from the sender rat. However, it should be considered that the behavior of the sender might be affected by the neural signal coming from the receiver (in other words if the sender rat will experience fewer seizures after the connection to the receiver – possibly because of diffusion of energy or extension of nervous system tissue). However, further research can be conducted to study this phenomenon. For example (1) a rat with depressive behavior connected to a rat with manic behavior or (2) a rat with insomnia connected to a rat with excessive sleep.

### Introducing the Notion of a Complex Biological Neural Code as a Way of Neuromodulation

An artificial spinal cord stimulation to treat chronic pain can be considered a simple stimulation paradigm. However, the neural code caused by seizures induced by PTZ coming from a sender rat can be regarded as a complex biological stimulation paradigm due to its nature and also due to a much bigger number of variables. Hence, further research can be done to study the effects of complex biological stimulation paradigms as possible new ways of neuromodulation.

## Other Possible Futuristic Applications of Spinal Cord – Spinal Cord Connection

**FIGURE 2:**
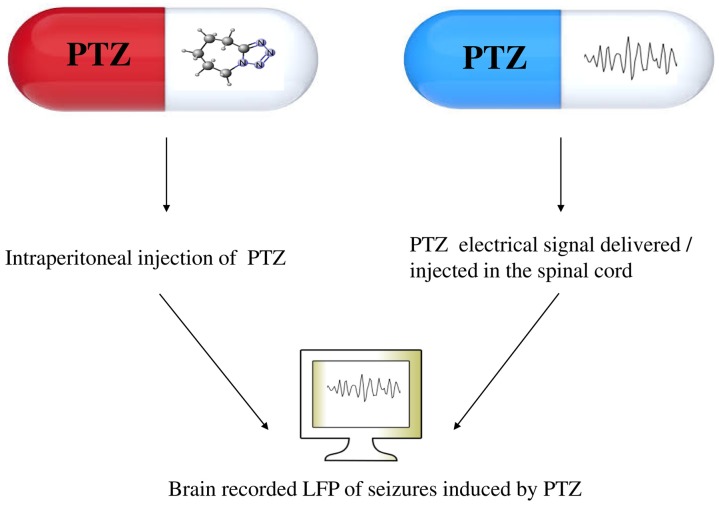
**The notion of an electrical medication**. The brain recorded LFP of seizures caused by the PTZ drug can also be caused by PTZ electrical activity delivered/injected in the spinal cord. The code or electrical activity of PTZ is what the author calls electrical drug. PTZ: pentylenetetrazol.

Of the several futuristic speculations, the author would like to highlight the notion of an electrical drug (**Figure 2**). For example, when PTZ is intraperitoneally injected on an animal, there will be seizure activity recorded on the brain LFP. If this neural activity (neuronal code) is injected (or delivered) in the spinal cord of a second animal, it is hypothesized that the second animal will have brain LFP that is equal to the LFP caused by the drug that is injected intraperitoneally.

**FIGURE 3:**
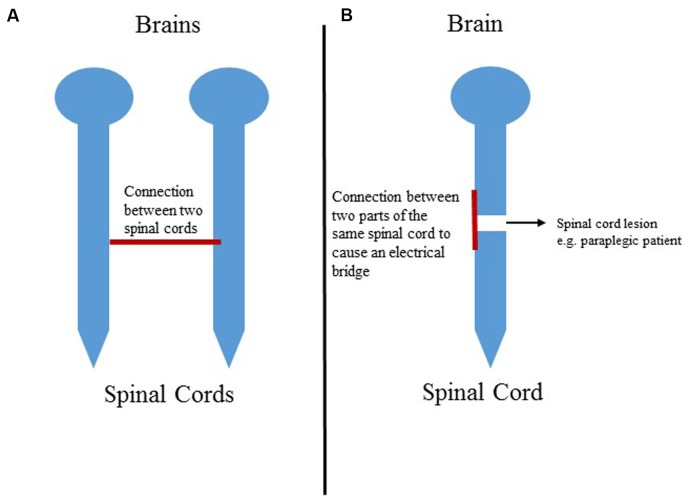
**(A)** Schematic of the connection between two spinal cords. **(B)** Representation of the connection between two parts of the same spinal cord in a paraplegic patient.

Another notion is that one can connect two parts of the same spinal cord in a paraplegic or tetraplegic patient to send information from the spinal cord portion below the lesion to the spinal cord segment that is above the segment (**Figure 3**). Moreover, this connection can be combined with other treatment approaches for paraplegic patients such as stem cells graft, walk again projects or spinal cord stimulation.

Regarding the applications of the notion of spinal cord – spinal cord connection to the fields of psychology, psychiatry and mental health, I speculate that, for example, a complex biological activity codifying relaxation can be recorded on an electronic device and then sent to the spinal cord of an individual with anxiety. On **Table 1** there is a list of the same rationale applied to modulate, study or treat different conditions such as anxiety, insomnia, bipolar disorder, schizophrenia, anorexia, obesity, and emotional instability due to personality disorder (just to name a few). Due to ethical issues, I do not consider possible applications of real-time spinal cord – spinal cord connection for psychiatric conditions in humans. Instead, the sender neural code should come from an electronic device.

**Table 1:** Possible applications of the knowledge related to spinal cord – spinal cord connection to psychology, psychiatry and mental health.

SENDER (Neural code recorded on an electronic device)	RECEIVER (Targeted individual or condition)
To promote relaxation	Anxiety
To promote relaxation	Sport performance
To induce sleep	Insomnia
To promote normal mood	Depressive phase of Bipolar Disorder as well as in Unipolar Major Depressive Disorder
To decrease pathologic elevated mood	Hypomanic or manic phase of Bipolar Disorder
To improve thought content and course	Psychotic conditions such as schizophrenia and schizoaffective disorder
To increase appetite	Anorexia
To decrease appetite	Obesity
To regulate emotions	Emotion instability in some types of personality disorders

## Conclusion

Spinal cord – spinal cord connection can be a new way of sharing information between two nervous systems. Complex codified electrical stimulation (such as the PTZ causing seizures) can be memorized and reproduced by the brain of a receiver. This principle may allow new methods to study neurophysiological process in the brain as well as new tools to model neuropsychiatric disorders. The connection of two spinal cords causes a shunt of information from an affected animal to a healthy one. This property can also be used therapeutically. The protocol proposed here can create a connection between two spinal cords that does not use amplifiers. A concept of electrical drug or electrical fingerprint of a drug is introduced. The notion of the connection between two parts of the same spinal cord to treat paraplegic or tetraplegic patients is also introduced. Possible applications of this technique to the fields of psychology, psychiatry and mental health. It can be said that information injected into the nervous system through the spinal cord as well as spinal cord – spinal cord connection can allow news ways of (1) studying neurophysiology, (2) modeling specific behaviors, (3) studying and modeling disease traits, (4) treating neuropsychiatric disorders and (5) information sharing between two nervous systems.

## Author Contributions

The author confirms being the sole contributor of this work and approved it for publication.

## Conflict of Interest Statement

The author declares that the research was conducted in the absence of any commercial or financial relationships that could be construed as a potential conflict of interest.
